# Sudden, Painless Incontinence following Extracorporeal Shock Wave Lithotripsy: A Case Report

**DOI:** 10.1155/2013/591543

**Published:** 2013-09-30

**Authors:** Fatih Akbulut, Alpaslan Akbas, Dilek Sahin

**Affiliations:** ^1^Department of Urology, Prof. Dr. Necmi Ayanoglu Silivri State Hospital, 34570 Silivri, Istanbul, Turkey; ^2^Department of Radiology, Prof. Dr. Necmi Ayanoglu Silivri State Hospital, Silivri, Istanbul, Turkey

## Abstract

Urinary incontinence is a stent-related complication; however, total incontinence is not often seen in emergency departments. We report a patient who presented with a sudden, painless, and total urinary incontinence after extracorporeal shock wave lithotripsy. This is the first case of total incontinence due to migrated ureteral double J stent through the external urethral sphincter into the urethra following extracorporeal shock wave lithotripsy.

## 1. Introduction

Ureteral double J stents have a high rate of complications. Urinary incontinence is a common stent-related complication, but especially total incontinence is not often seen in emergency departments. We describe a patient who presented with a sudden, painless, and total urinary incontinence after extracorporeal shock wave lithotripsy (ESWL). 

## 2. Case Report

**Figure 1:**
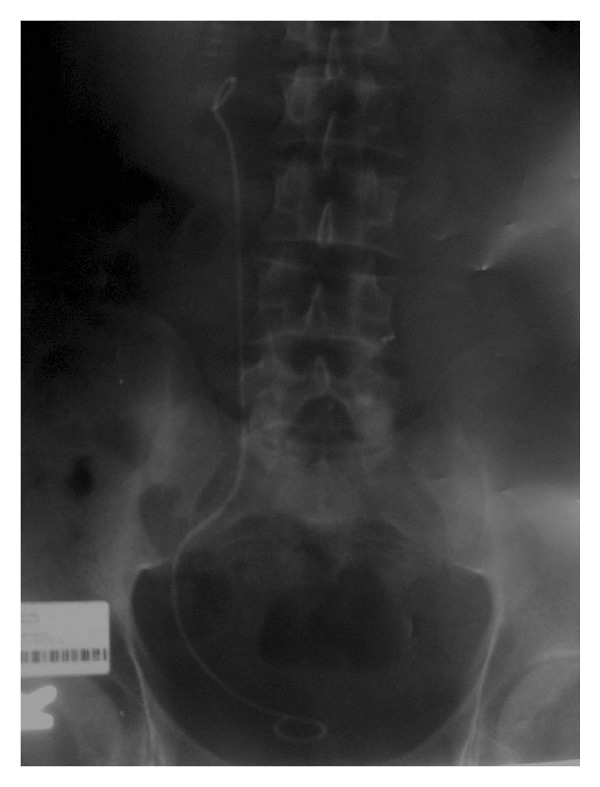
Kidney, ureter, and bladder (KUB) X-ray showing proper stent placement.

A 25-year-old male prisoner with a 3-day history of right lumbar pain presented to our clinic. Kidney, ureter, and bladder (KUB) X-ray, ultrasonography, and CT scan revealed a 1.5 cm right proximal ureteral stone with hydronephrosis. Renal laboratory parameters and urinalysis were normal. We performed a right ureterorenoscopy but were unable to perform lithotripsy due to ureteral edema and an impacted stone. We simultaneously placed a double J stent during KUB X-ray (Figure 1). He was then referred to an education and research hospital for ESWL.

He presented to our emergency department with a sudden, painless, and total incontinence two days after the first ESWL session and was referred to our urology department. He said that he had no problems after his ESWL session until he noticed that his underwear was wet. He changed his underwear but continued to experience incontinence during the day.

**Figure 2:**
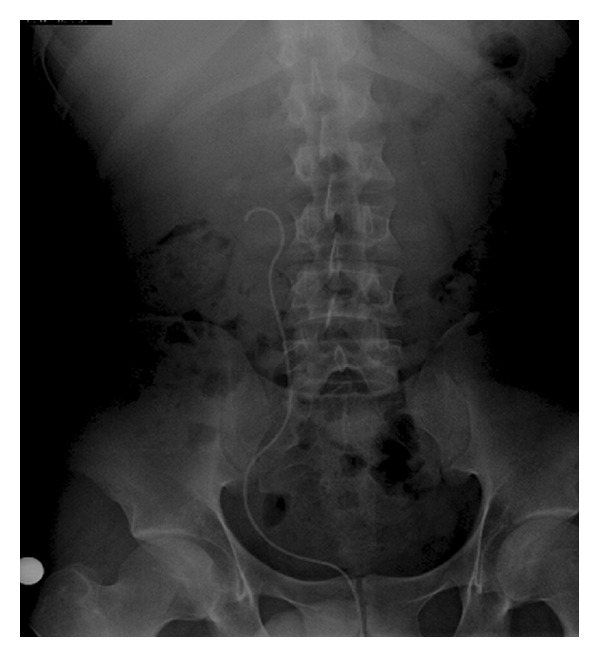
Kidney, ureter, and bladder (KUB) X-ray study showing migration of the double J stent into the urethra after extracorporeal shock wave lithotripsy (ESWL).

The patient had an unremarkable medical history and used anti-inflammatory drugs for renal stones. He experienced anxiety, excitement, and embarrassment because he lived in a jail cell with other prisoners. His vital signs were stable and he did not have abdominal or costovertebral angle tenderness upon physical examination. There were no genital abnormalities. Urinalysis and a KUB X-ray study were performed, which demonstrated that the double J stent had migrated into the urethra (Figure 2). To our knowledge, the patient's total incontinence was the result of the stent bypassing the urethral sphincteric mechanism.

The stent was found in the membranous urethra and was removed cystoscopically under local anesthesia. His incontinence remitted immediately after the stent was removed. We suggested that he continue with ESWL, but he was lost to follow-up. This is the first case of sudden total incontinence due to migrated ureteral double J stent through the external urethral sphincter into the urethra following ESWL.

## 3. Discussion

Since the first report of double J ureteral stent use in 1967 by Zimskind et al [1], its design has undergone many improvements. However, complications associated with double J ureteral stents remain common. 

Stent-related symptoms can vary from one patient to another. Several studies have reported that the most common stent-related symptoms are incomplete emptying (76%), urgency (57–60%), frequency (50–60%), dysuria (40%), suprapubic (30%), flank pain (19–32%), urinary incontinence, and hematuria (25%) [2–9]. 

The main types of incontinence are urge, stress, mixed and continuous. Urge incontinence is defined as the involuntary loss of urine triggered by a strong desire to immediately void the bladder. Stent-related urge incontinence begins with urgency symptoms, and it is also possible that stent placement may unmask or exacerbate preexisting, subclinical detrusor instability [9]. 

Total incontinence is continuous leaking of urine, day and night, or the periodic uncontrollable leaking of large volumes of urine. It has congenital, iatrogenic, and neurogenic causes. Stent-related total incontinence is the result of stent migration along the bladder to the distal urethra, bypassing the external urethral sphincter [9]. Sengottayan et al. described the first case of a patient with continuous incontinence due to the fragmentation of a forgotten double J ureteral stent across the urethral sphincter [10]. Delasobera and Rogers reported incontinence in a patient with a history of urolithiasis, double J stent, and nephrostomy tube placement. Radiologic evaluation revealed that her stent had crossed the urethra. It was removed without difficulty, and the patient's incontinence was immediately resolved [11]. In our case, double J stent placement and ESWL were performed because of a proximal ureter stone. The patient presented with sudden, total incontinence following his first ESWL session. Urethral migration was predicted because of his history and was confirmed with KUB X-ray.

In conclusion, urinary incontinence is commonly seen in patients with ureteral stents, but sudden, painless, and total incontinence is very rare, and there is no study regarding its prevalence. Stent migration to the urethra should be considered when a patient with a history of ureteral catheterization presents with a sudden, painless and total incontinence.
